# Immediate Effects of Leg-Press Coordination Training on Ankle Sway in Individuals With Chronic Ankle Instability: A Randomized Controlled Trial

**DOI:** 10.7759/cureus.72335

**Published:** 2024-10-24

**Authors:** Ryo Miyachi, Yui Nagamori, Yoshinari Fujii, Yuji Kanazawa

**Affiliations:** 1 Faculty of Health and Medical Sciences, Hokuriku University, Kanazawa, JPN

**Keywords:** ankle joint, coordination, instability, leg-press, training

## Abstract

Objectives

This study aimed to determine the effects of leg-press coordination training on immediate ankle sway in individuals with chronic ankle instability (CAI).

Methods

Participants with CAI (age 19.8 ± 1.0 years, seven men and 17 women) were randomly allocated to a control group (CON), which performed regular leg-press training, or a coordination training group (CT), which performed coordination training using a leg-press device. The main outcome measure was the average angular jerk cost of the ankle joint in the Y-balance test (YBT), and the secondary outcome measures were the maximum ankle and hip joint angles, maximum reach distance in the YBT, ankle proprioception, and weight-bearing dorsiflexion angle.

Results

A significant group × period (pre- and post-intervention) interaction in the ankle average angular jerk cost was observed in the YBT in anterior reaching and posteromedial reaching ankle plantar flexion/dorsiflexion (anterior reaching: p = 0.03, posteromedial reaching: p < 0.01) as well as in adduction/abduction (posteromedial reaching: p = 0.02). The average ankle angular jerk cost in the CT group was significantly lower at post-intervention than at pre-intervention.

Conclusions

Leg-press coordination training immediately reduces ankle sway in individuals with CAI.

## Introduction

Chronic ankle instability (CAI) is a frequent sequela of ankle sprains, and interventions to reduce ankle sway caused by CAI are required. Ankle sprains account for 25% of sports injuries [1], and approximately 40% of patients develop CAI within 12 months of sustaining a sprain injury [2]. CAI results in symptoms such as pain, weakness, and limited range of motion around the ankle joint owing to instability and swaying of the ankle joint, thus leading to repeated ankle sprains [3]. Continuous tissue overload and repetitive ankle sprains associated with ankle sway promote ankle instability and increase the risk of developing ankle osteoarthritis because of structural failure of the ankle joint [4,5]. Therefore, early intervention to reduce ankle sway associated with CAI is important.

Coordination training is often used to reduce ankle sway. A high level of active control by muscle contraction is required when the stability of noncontractile tissue is defective, such as after trauma [6]. Moreover, coordination training enhances active control by improving spinal reflex modulation, corticospinal excitability, proprioception, muscle reaction speed, and changing the recruitment patterns of muscle groups [7-10]. Therefore, coordination training, including postural balance training, is often used to enhance active control and reduce ankle sway in patients with CAI [11-14].

However, coordination training has several limitations and may be difficult for some individuals to implement. It is often performed in an anti-gravity position, occasionally on an unstable floor surface, using static/dynamic postural-holding tasks. Falls and sudden joint motions may easily cause recurrent sprains, and load adjustment may be difficult. Therefore, a safe, versatile, and effective method of coordination training is required.

Leg-press training has long been utilized to strengthen the muscles of the lower limbs in various people because of its simplicity, lack of risk of falling, and advantage of easily adjusting the amount of load [15,16]. Adding a coordination component to leg-press training will safely reduce ankle sway. However, no studies have investigated the effect of leg press-based coordination training on the reduction of ankle sway, especially those verified using the kinematic parameters of ankle sway. The effect of immediate reduction of ankle sways as a warm-up before sports activities and other activities may contribute to reducing the load on the ankle joint in CAI and preventing recurrent sprains.

Therefore, this study aimed to clarify the immediate effect of less-press coordination training on the reduction of ankle sway in patients with CAI using kinematic parameters. The hypothesis was that coordination training with a leg-press device would immediately reduce ankle sway.

## Materials and methods

Study design

In this single-blind, randomized controlled trial, participants were randomly assigned to a control group (CON) that performed regular leg-press training using a leg-press device and a coordination training group (CT) that used a leg-press device. Stratified randomization was performed using a random number table in Microsoft Excel (Microsoft Corp., Redmond, WA) to ensure equal proportions of men and women in each group. Measurements and data analyses regarding group allocation were performed blindly. Only one intervention session was conducted, and each outcome was measured immediately pre- and post-intervention.

Participants and setting

This study enrolled university students ages 18-25 on a basketball team (top-level local team) who volunteered to participate in September 2023 and who had CAI and did not meet the inclusion/exclusion criteria. Informed consent was obtained from 59 participants (22 men and 37 women, with an average age, height, and weight of 19.7 ± 1.0 years, 170.8 ± 9.0 cm, and 63.7 ± 8.7 kg, respectively), of whom 24 with CAI (12 in the CON group and 12 in the CT group) were eligible for this study. The criteria for CAI were as follows: history of at least one ankle sprain; repeated ankle joint pain, swelling, and instability; repeated giving way; and a Cumberland Ankle Instability Tool (CAIT) score of ≤25, according to a previous study [17]. The exclusion criteria were as follows: acute ankle sprain within one month after injury, history of fracture around the ankle joint, history of lower limb or trunk surgery, pain that interferes with daily life except in the ankle joint, and typical physical disability, such as paralysis due to cerebrovascular disease. The leg on the side that met the abovementioned CAI conditions (in the bilateral case, the leg with the lower CAIT score) was measured. The study was conducted from September 2023 to February 2024. All measurements and training were performed in a laboratory at the authors’ institution. Test registration date: February 1, 2024. Participant recruitment: November 1, 2023-February 10, 2024. Data collection: February 2, 2024-February 19, 2024.

Ethical approval

The study was conducted in accordance with the principles of the Declaration of Helsinki and approved by the Ethics Committee of our institution (approval no. 2023-1). Written informed consent was obtained from the participants before study initiation. This study was conducted after registration with the University Hospital Medical Information Network Clinical Trials Registry (UMIN000053503) as a clinical trial.

Intervention

**Figure 1:**
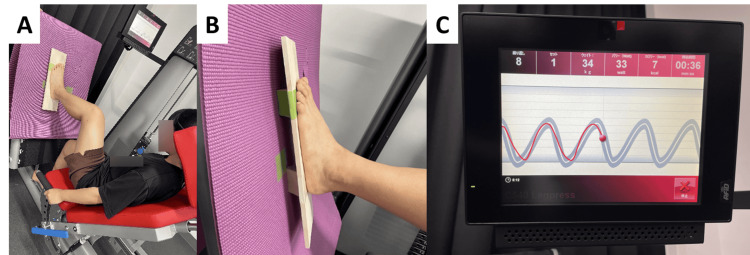
Coordinated training methods using leg presses

Training was performed using a leg-press device (Compass 540, SAKAI Medical Co., Ltd., Tokyo, Japan), and only one session of the one-leg leg press exercise with the test leg was performed immediately after the pre-intervention measurement. The starting position was the plantar foot of the test leg positioned with the tibia perpendicular to the footplate of the device. The chair position was adjusted such that the knee joint was in a 90° flexed position. The backrest was placed at a 45° inclination to the floor. The load was set at 50% of the body weight, assuming a one-leg standing posture, with three sets of 10 repetitions and a 45-second rest between sets. Training was limited to mild knee flexion to avoid full extension of the knee joint during leg extension. The CON group performed leg-press exercises at the rhythm of the participants’ choice on the normal plantar surface. In the CT group, the plantar surface of the participant's leg was placed on a balance board that was prone to sway in the inversion/eversion direction, and leg-press exercises were performed while striving to maintain the ankle joint in a neutral position on the forehead plane during the training (Figure 1).

In addition, the leg-press exercise was performed with visual feedback according to the timing of lower extremity flexion displayed on the attached monitor (Figure 1). Training was always supervised by one co-author to ensure no errors in the training content.

Outcome measurement

**Figure 2:**
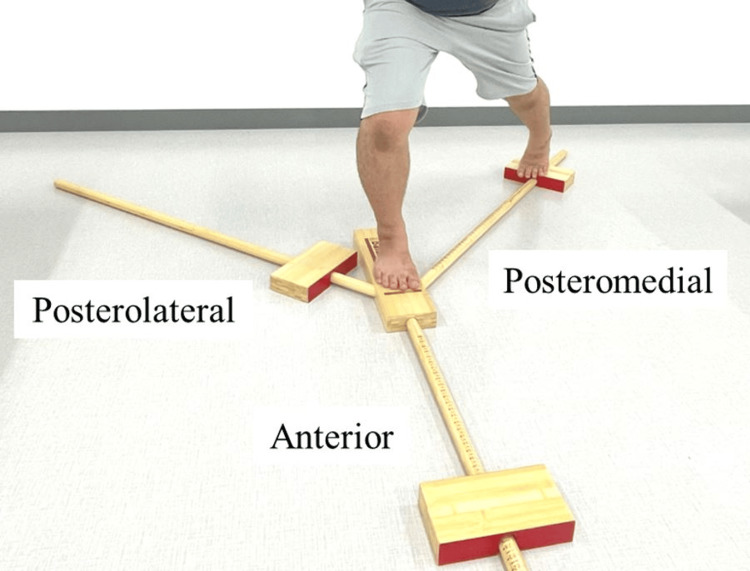
Y-balance test

The main outcome measure was the average angular jerk cost of the ankle joint in the Y-balance test (YBT) as a measure of ankle sway. The secondary outcome measures were the maximum reach distance and maximum ankle and hip joint angles in the YBT, passive joint repositioning sense (PJRS) of the ankle joint as a measure of ankle proprioception, and weight-bearing dorsiflexion angle. All measurements were taken barefoot. In the YBT, the average ankle angular jerk cost, maximum ankle joint angle, and maximum reach distance were measured during leg reach, with the leg on the measurement side as the supporting leg and the contralateral leg as the reaching leg. An increase in the angular jerk cost implies rapid acceleration/deceleration of the joint, and a large angular jerk cost can be interpreted as a large joint sway. The direction of reach was determined as anterior, posteromedial, or posterolateral, based on the reach leg (Figure 2).

A YBT Kit (Perform Better, West Warwick, Rhode Island, USA) was used for the measurements. In the YBT, the block in each direction was moved as far as possible with the toe of the reaching leg, while the plantar of the supporting leg remained on the floor, and then returned to the starting position. While reaching, the palms were positioned at the waist, and the gaze was not specified but could be used freely for balancing. Demonstrations and multiple practice sessions were conducted before the measurements were obtained to ensure that the participants understood the movement method and that their movements were stable. Task failure was defined as the inability to hold a one-legged standing position, the heel or toe of the supporting leg leaving the floor, the toe of the reaching leg completely touching the floor, or the inability to return to the starting position after reaching. In the case of failure, successful attempts were remeasured and included in the analysis. The maximum reach distance was standardized (reach distance / leg length × 100) by dividing the obtained value by the participant's leg length (trochanter malleolar distance) and was used as a measure of the maximum reach distance. A small accelerometer (AMWS020, ATR-Promotions, Sagara, Japan) and receiver software (Sensor Controller, ATR-Promotions, Sagara, Japan) were used to measure the kinematic parameters of the YBT. The small accelerometer was fixed directly to the skin with tape at four locations on the test side: the center of the sacrum, the center of the posterior thigh, the center of the posterior lower leg, and the heel (back of the calcaneus) of the lower limb. The acceleration range was set to ±8 G, the angular rate range to ±1.000 dps, and the sampling frequency to 100 Hz to acquire sensor tilt angular velocity data. In the data analysis, the angular jerk cost (∫t₀ᵗ [(dθ³/dt³)²] dt) of the ankle joint was calculated from the angular velocity data of the sensors, and the average value was used [18]. The maximum ankle and hip joint angles were calculated using the angular velocity data obtained from the sensors. Ankle plantar flexion and hip extension angles were excluded from the analysis because the maximum joint angle was at the starting or ending position. The measurements were performed in two trials in each of the three directions, and the average of the two measurements was used as the representative value.

To measure the PJRS of the ankle joint, the participants placed their feet on a handmade device in a sitting position with their eyes closed, and 15° plantar flexion or 15° inversion of the ankle joint was presented beforehand. Subsequently, the target position was set by passive motion from the neutral ankle position, and the angle was measured [19]. The measurements were performed three times, and the absolute error and variable error of the three measurements were calculated [20].

For the weight-bearing dorsiflexion angle, the test leg was stepped forward. The foot was positioned such that the heel and second toe were aligned, and the lower leg was tilted forward with the heel grounded. An angle meter was placed at the center of the tibia 15 cm below the rough tibial surface to measure the angle of inclination of the lower leg.

Statistics analyses

All statistical analyses were performed using the SPSS version 28 (IBM SPSS Statistics for Windows, IBM Corp., Armonk, NY). The sample size was calculated using G*Power v.3.1.9.7 (Heinrich Heine University, Düsseldorf, Germany) with an effect size of 0.4, an alpha of 0.05, and a power of 0.8, resulting in 28 participants (14 in each group). Chi-square and unpaired t-tests were used for between-group comparisons of general characteristics and pre-intervention values. Each outcome was compared by analyzing variance (group × time) using a split-plot design with pre-intervention values as covariates and a simple main-effects test. The significance level was set at p < 0.05.

## Results

General characteristics of the participants

**Figure 3:**
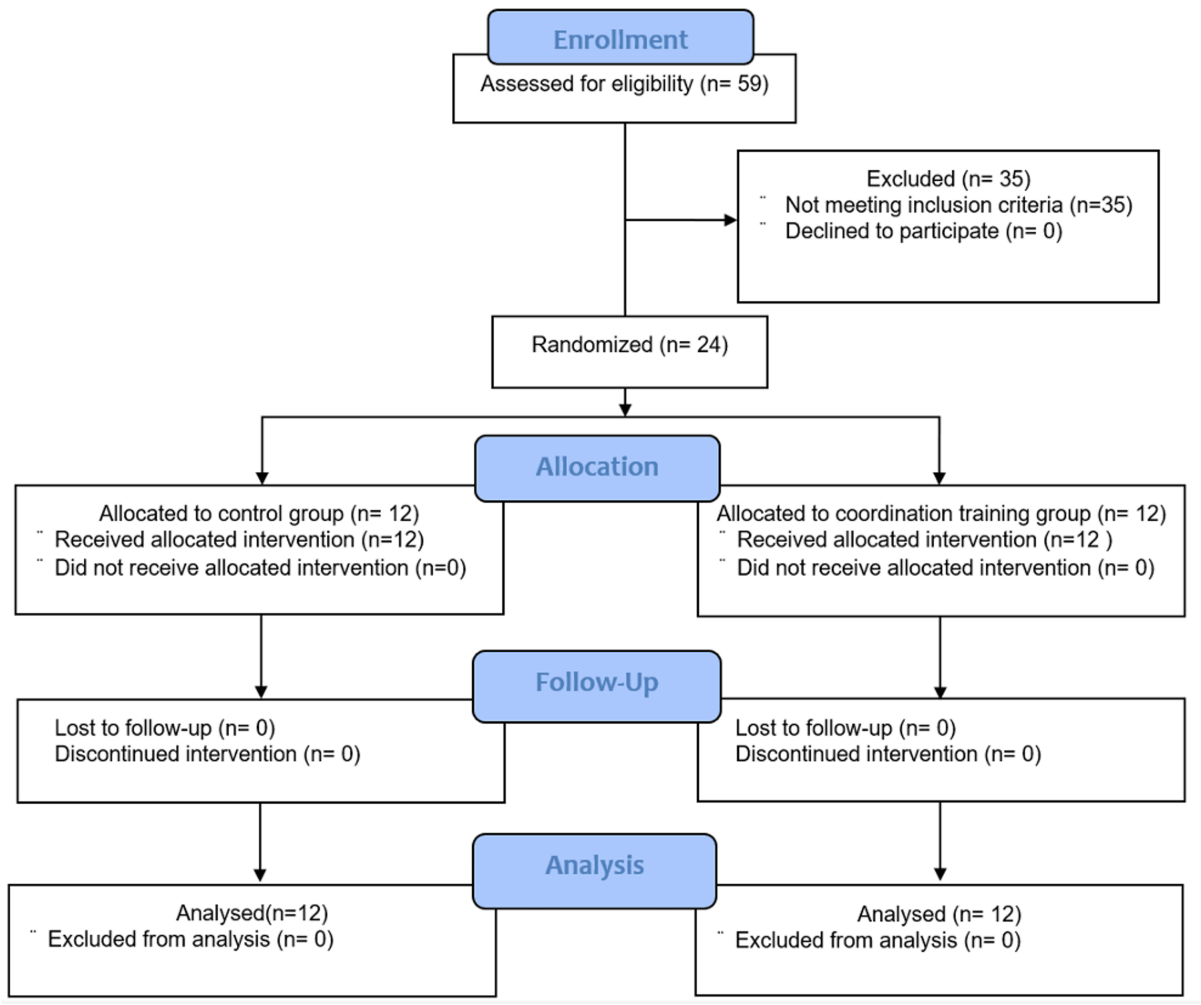
Participant flowchart

A flowchart on participant selection is shown in Figure 3. In total, 24 participants who met the inclusion criteria were included in the final analysis. Their general characteristics are presented in Table 1.

**Table 1:** General characteristics of the participants Values are presented as the number of participants or mean (standard deviation). CAIT, Cumberland Ankle Instability Tool; CON, control; CT, coordination training

Characteristic	Overall (N = 24)	CON group (N = 12)	CT group (N = 12)	p-value
Sex (N)	Male, 7; female, 17	Male, 3; female, 9	Male, 4; female, 8	0.65
Age (years)	19.8 (1.0)	19.5 (1.0)	20.2 (0.9)	0.11
Height (cm)	170.1 (9.5)	170.3 (10.5)	169.9 (8.9)	0.93
Weight (kg)	63.5 (9.5)	63.3 (10.2)	63.7 (9.2)	0.91
Exercise time per week (hours)	17.3 (4.0)	16.0 (3.5)	18.5 (4.2)	0.13
CAIT (score)	19.2 (5.3)	18.5 (6.3)	19.9 (4.3)	0.53

Age, sex, height, weight, weekly exercise duration, and CAIT on the participants’ side were not significantly different between the CON and CT groups.

Main outcome measure

The pre-intervention values of the average ankle angular jerk cost in the YBT were not significantly different between the two groups in all planes of motion and in the direction of reach (Table 2).

**Table 2:** Outcome measure results in the pre-intervention period Values are presented as means (standard deviations). *Significant difference (p < 0.05). CON, control; CT, coordination training; PJRS, passive joint repositioning sense; YBT, Y-balance test

Outcome measure				CON group (N = 12)	CT group (N = 12)	p-value
YBT	Anterior	Reach distance / leg length (%)		48.3 (6.3)	47.4 (5.8)	0.71
		Average ankle joint angular jerk cost	Inversion and Eversion (deg^2^/sec^5^)	4.5 × 10^7^ (3.0 × 10^7^)	3.2 × 10^7^ (1.8 × 10^7^)	0.23
			Plantar and dorsiflexion (deg^2^/sec^5^)	5.1 × 10^7^ (4.7 × 10^7^)	4.5 × 10^7^ (2.7 × 10^7^)	0.71
			Adduction and Abduction (deg^2^/sec^5^)	2.3 × 10^8^ (2.3 × 10^8^)	1.7 × 10^8^ (9.4 × 10^7^)	0.40
		Maximum ankle joint angle	Dorsiflexion (deg)	23.7 (3.7)	22.4 (6.1)	0.55
			Eversion (deg)	5.3 (5.0)	6.2 (4.2)	0.65
			Inversion (deg)	3.7 (2.4)	3.5 (3.2)	0.90
			Adduction (deg)	4.6 (3.3)	4.0 (2.8)	0.61
			Abduction (deg)	6.3 (4.7)	6.1 (2.2)	0.91
		Maximum hip joint angle	Flexion (deg)	37.4 (13.1)	40.5 (15.1)	0.59
			External rotation (deg)	12.7 (10.5)	8.6 (15.6)	0.45
			Internal rotation (deg)	11.4 (12.3)	19.0 (12.8)	0.15
			Adduction (deg)	7.7 (5.5)	2.9 (4.7)	0.03*
			Abduction (deg)	8.1 (3.1)	9.7 (4.9)	0.36
	Posterolateral	Reach distance / leg length (%)		80.2 (10.6)	76.1 (8.2)	0.30
		Average ankle joint angular jerk cost	Inversion and Eversion (deg^2^/sec^5^)	6.0 × 10^7^ (3.7 × 10^7^)	9.0 × 10^7^ (1.3 × 10^8^)	0.46
			Plantar and dorsiflexion (deg^2^/sec^5^)	7.6 × 10^7^ (5.3 × 10^7^)	8.7 × 10^7^ (7.1 × 10^7^)	0.66
			Adduction and Abduction (deg^2^/sec^5^)	3.5 × 10^8^ (3.3 × 10^8^)	2.9 × 10^8^ (1.8 × 10^8^)	0.60
		Maximum ankle joint angle	Dorsiflexion (deg)	23.7 (3.7)	22.4 (6.1)	0.55
			Eversion (deg)	5.3 (5.0)	6.2 (4.2)	0.65
			Inversion (deg)	3.7 (2.4)	3.5 (3.2)	0.90
			Adduction (deg)	4.6 (3.3)	4.0 (2.8)	0.61
			Abduction (deg)	6.3 (4.7)	6.1 (2.2)	0.91
		Maximum hip joint angle	Flexion (deg)	77.4 (11.2)	76.1 (17.3)	0.84
			External rotation (deg)	19.7 (13.2)	14.3 (17.1)	0.40
			Internal rotation (deg)	15.5 (13.1)	23.0 (10.7)	0.14
			Adduction (deg)	16.3 (15.9)	22.9 (11.8)	0.26
			Abduction (deg)	22.5 (17.1)	10.0 (15.2)	0.07
	Posteromedial	Reach distance / leg length (%)		87.7 (8.1)	89.6 (10.0)	0.61
		Average ankle joint angular jerk cost	Inversion and Eversion (deg^2^/sec^5^)	5.1 × 10^7^ (3.6 × 10^7^)	4.3 × 10^7^ (1.7 × 10^7^)	0.50
			Plantar and dorsiflexion (deg^2^/sec^5^)	5.5 × 10^7^ (4.5 × 10^7^)	3.9 × 10^7^ (1.5 × 10^7^)	0.28
			Adduction and Abduction (deg^2^/sec^5^)	2.3 × 10^8^ (1.7 × 10^8^)	2.1 × 10^8^ (1.0 × 10^8^)	0.63
		Maximum ankle joint angle	Dorsiflexion (deg)	23.7 (3.7)	22.4 (6.1)	0.55
			Eversion (deg)	5.3 (5.0)	6.2 (4.2)	0.65
			Inversion (deg)	3.7 (2.4)	3.5 (3.2)	0.90
			Adduction (deg)	4.6 (3.3)	4.0 (2.8)	0.61
			Abduction (deg)	6.3 (4.7)	6.1 (2.2)	0.91
		Maximum hip joint angle	Flexion (deg)	90.4 (11.7)	89.1 (18.0)	0.83
			External rotation (deg)	18.2 (15.2)	9.0 (12.6)	0.12
			Internal rotation (deg)	12.2 (13.4)	20.8 (14.8)	0.15
			Adduction (deg)	10.0 (7.6)	4.8 (5.7)	0.07
			Abduction (deg)	10.6 (10.1)	24.2 (14.4)	0.01*
PJRS	Inversion		Absolute error (deg)	2.2 (1.1)	1.4 (0.9)	0.09
			Variable error (deg)	1.1 (0.8)	1.0 (0.3)	0.66
	Plantar flexion		Absolute error (deg)	2.2 (1.3)	1.9 (1.1)	0.55
			Variable error (deg)	1.3 (0.8)	1.4 (0.8)	0.92
Weight-bearing dorsiflexion angle (deg)				42.3 (7.4)	41.8 (7.9)	0.87

The average ankle angular jerk cost in the YBT showed a significant group × period (pre- and post-intervention) interaction in ankle plantar flexion/dorsiflexion during anterior reaching and in ankle plantar flexion/dorsiflexion and adduction/abduction during posteromedial reaching (Table 3).

**Table 3:** Main outcome measurement results post-intervention *Significant interaction or difference (p < 0.05). CON; control; CT, coordination training

Outcome measure		CON group (N = 12)		CT group (N = 12)		Group and time interaction		Main effect	
		Mean (standard deviation) (deg^2^/sec^5^)	p-value for pre-/post-comparisons	Mean (standard deviation) (deg^2^/sec^5^)	p-value for pre-/post-comparisons	p-value	Effect size (partial η^2^)	p-value	Effect size (partial η^2^)
Anterior	Inversion and eversion	6.5 × 10^7^ (7.6 × 10^7^)	0.74	3.3 × 10^7^ (2.0 × 10^7^)	0.25	0.44	0.03	0.78	<0.01
	Plantar and dorsiflexion	1.1 × 10^8^ (1.1 × 10^8^)	0.44	3.8 × 10^7^ (1.4 × 10^7^)	<0.01*	0.03*	0.20	0.46	0.03
	Adduction and abduction	2.8 × 10^8^ (2.0 × 10^8^)	0.05	1.9 × 10^8^ (1.5 × 10^8^)	0.80	0.42	0.03	0.05*	0.18
Posterolateral	Inversion and eversion	6.0 × 10^7^ (3.6 × 10^7^)	0.30	5.0 × 10^7^ (2.4 × 10^8^)	<0.01*	0.26	0.06	<0.01*	0.64
	Plantar and dorsiflexion	8.5 × 10^7^ (3.6 × 10^7^)	0.01*	5.7 × 10^7^ (4.2 × 10^7^)	0.05*	0.05	0.17	<0.01*	0.44
	Adduction and abduction	3.7 × 10^8^ (2.3 × 10^8^)	0.02*	2.3 × 10^8^ (1.2 × 10^8^)	0.05*	0.05	0.17	<0.01*	0.34
Posteromedial	Inversion and eversion	5.2 × 10^7^ (4.3 × 10^7^)	0.76	3.6 × 10^7^ (1.8 × 10^7^)	0.42	0.37	0.04	0.72	<0.01
	Plantar and dorsiflexion	6.3 × 10^7^ (3.3 × 10^7^)	0.01*	3.4 × 10^7^ (1.3 × 10^7^)	0.09	0.01*	0.27	<0.01*	0.38
	Adduction and abduction	2.9 × 10^8^ (1.7 × 10^8^)	0.08	1.6 × 10^8^ (9.2 × 10^7^)	0.14	0.02*	0.24	0.05	0.17

In the pre- and post-intervention comparisons in the CON group, ankle plantar flexion/dorsiflexion and adduction/abduction during posterolateral reaching and ankle plantar flexion/dorsiflexion during posteromedial reaching were significantly higher post-intervention than pre-intervention. The post-intervention values were significantly lower than the pre-intervention values for ankle plantar flexion/dorsiflexion during anterior reaching and for all motion directions during posterolateral reaching (Table 3).

Secondary outcome measures

The pre-intervention values for the maximum reach distance and maximum ankle joint angle in the YBT, absolute and variable errors in the PJRS, and weight-bearing dorsiflexion angle were not significantly different between the two groups. The maximum hip joint angle in the YBT was significantly different only for adduction during the anterior reach and abduction in the posteromedial reach (Table 2). Only the maximum hip adduction angle for the anterior reach in the YBT showed a significant group × period interaction; however, no significant group × period interactions were observed for the maximum reach distance and maximum ankle joint angle in the YBT, absolute error and variable error in the PJRS, or weight-bearing dorsiflexion angle (Table 4).

**Table 4:** Secondary outcome measurement results post-intervention Values are presented as means (standard deviations). *Significant interaction or difference (p < 0.05). CON, control; CT, coordination training; PJRS, passive joint repositioning sense; YBT, Y-balance test

Outcome measure				CON group (N = 12)		CT group (N = 12)		Group and time interaction		Main effect	
				Mean (standard deviation) (deg^2^/sec^5^)	p-value for pre-/post-comparisons	Mean (standard deviation) (deg^2^/sec^5^)	p-value for pre-/post-comparisons	p-value	Effect size (partial η^2^)	p-value	Effect size (partial η^2^)
YBT	Anterior	Reach distance / leg length (%)		48.6 (6.3)	0.44	46.6 (5.5)	0.31	0.28	0.05	0.20	0.08
		Maximum ankle joint angle	Dorsiflexion (deg)	24.0 (4.2)	0.42	21.3 (5.7)	0.34	0.19	0.08	0.16	0.09
			Eversion (deg)	6.1 (4.2)	0.03*	6.2 (5.0)	0.78	0.52	0.02	0.18	0.08
			Inversion (deg)	3.5 (1.9)	0.07	3.3 (3.3)	0.95	0.89	<0.01	0.29	0.05
			Adduction (deg)	3.8 (3.6)	0.79	3.9 (2.6)	0.19	0.56	0.02	0.30	0.05
			Abduction (deg)	6.7 (4.8)	0.44	5.7 (3.6)	0.93	0.46	0.03	0.69	<0.01
		Maximum hip joint angle	Flexion (deg)	40.2 (9.5)	0.01*	35.2 (14.6)	0.65	0.03*	0.21	0.04*	0.19
			External rotation (deg)	12.0 (11.3)	0.75	7.0 (9.9)	0.23	0.37	0.04	0.16	0.09
			Internal rotation (deg)	12.8 (14.5)	0.92	17.0 (11.7)	0.48	0.30	0.05	0.68	0.01
			Adduction (deg)	8.1 (4.4)	<0.01*	3.4 (4.2)	0.05*	0.02*	0.24	<0.01*	0.50
			Abduction (deg)	6.1 (3.6)	0.83	9.7 (4.9)	0.21	0.08	0.14	0.24	0.07
	Posterolateral	Reach distance / leg length (%)		81.4 (11.9)	0.75	78.9 (9.8)	0.30	0.76	<0.01	0.33	0.05
		Maximum ankle joint angle	Dorsiflexion (deg)	17.6 (4.5)	0.03*	15.5 (5.5)	0.04*	0.46	0.03	<0.01*	0.36
			Eversion (deg)	5.4 (3.4)	0.50	5.8 (3.7)	0.15	0.44	0.03	0.06	0.16
			Inversion (deg)	4.3 (3.2)	0.94	3.2 (2.0)	0.21	0.69	0.01	0.52	0.02
			Adduction (deg)	6.1 (3.1)	0.36	6.4 (4.2)	0.14	0.81	<0.01	0.04*	0.19
			Abduction (deg)	6.9 (3.9)	0.20	7.5 (4.0)	0.32	0.61	0.01	0.84	0.14
		Maximum hip joint angle	Flexion (deg)	73.9 (12.7)	0.61	75.9 (10.5)	<0.01*	0.48	0.02	<0.01*	0.35
			External rotation (deg)	20.8 (12.7)	0.50	12.3 (13.9)	0.15	0.06	0.16	0.10	0.13
			Internal rotation (deg)	15.4 (14.3)	0.83	23.0 (11.9)	0.55	0.83	<0.01	0.76	0.01
			Adduction (deg)	17.6 (17.7)	0.86	19.2 (14.1)	0.63	0.12	0.11	0.56	0.02
			Abduction (deg)	21.1 (19.5)	0.67	14.1 (17.2)	0.16	0.09	0.13	0.78	<0.01
	Posteromedial	Reach distance / leg length (%)		87.9 (8.0)	0.39	87.2 (10.0)	0.73	0.16	0.09	0.39	0.04
		Maximum ankle joint angle	Dorsiflexion (deg)	17.9 (3.9)	0.03*	18.2 (4.5)	0.10	0.65	0.01	<0.01*	0.31
			Eversion (deg)	5.5 (2.9)	0.10	5.6 (4.6)	0.39	0.95	<0.01	0.09	0.13
			Inversion (deg)	2.8 (2.0)	0.13	3.2 (2.7)	0.33	0.52	0.02	0.10	0.13
			Adduction (deg)	3.9 (3.5)	0.96	4.5 (1.9)	0.03*	0.54	0.02	0.18	0.08
			Abduction (deg)	6.6 (2.4)	0.02*	5.3 (2.4)	0.03*	0.37	0.04	<0.01*	0.40
		Maximum hip joint angle	Flexion (deg)	86.7 (10.7)	0.12	87.5 (15.8)	0.12	0.67	0.01	0.03*	0.21
			External rotation (deg)	16.1 (13.3)	0.57	8.1 (10.9)	0.58	0.76	<0.01	0.40	0.03
			Internal rotation (deg)	11.6 (12.4)	0.57	20.6 (13.0)	0.18	0.40	0.03	0.14	0.10
			Adduction (deg)	10.0 (9.3)	0.57	4.8 (7.7)	0.69	0.72	0.01	0.48	0.02
			Abduction (deg)	9.5 (13.1)	0.16	22.1 (12.5)	0.34	0.92	<0.01	0.97	<0.01
PJRS	Inversion		Absolute error (deg)	1.4 (0.6)	0.02*	1.8 (0.9)	0.01*	0.28	0.06	<0.01*	0.25
			Variable error (deg)	0.9 (0.6)	0.01*	1.1 (0.8)	0.12	0.53	0.02	<0.01*	0.34
	Plantar flexion		Absolute error (deg)	1.4 (0.8)	0.01*	1.4 (0.6)	<0.01*	0.97	<0.01	<0.01*	0.60
			Variable error (deg)	0.8 (0.4)	<0.01*	1.1 (0.5)	<0.01*	0.21	0.07	<0.01*	0.65
Weight-bearing dorsiflexion angle (deg)				43.8 (6.4)	0.07	43.0 (7.8)	0.36	0.79	<0.01	0.05*	0.18

Significant differences in the maximum ankle joint angles in the YBT for eversion during the anterior reach, dorsiflexion during the posterolateral reach, and dorsiflexion and abduction during the posteromedial reach were observed in the CON group between the pre- and post-intervention periods. The maximum hip joint angles pre- and post-intervention were significantly different for flexion and adduction during anterior reach. In the PJRS, absolute and variable errors for both inversion and plantar flexion were significantly lower post-intervention than pre-intervention (Table 4). Significant differences in the maximum ankle joint angle in the YBT for dorsiflexion during the posterolateral reach and adduction and abduction during the posteromedial reach were observed in the CT group between the pre-intervention and post-intervention periods. Significant differences in the maximum hip joint angle were observed in adduction during the anterior reach and in flexion during the posterolateral reach pre- and post-intervention. In the PJRS, the absolute error of inversion was significantly higher at post-intervention than at pre-intervention, and the absolute and variable errors of plantar flexion were significantly lower at post-intervention than at pre-intervention (Table 4).

## Discussion

To the best of our knowledge, this is the first study to use kinematic parameters to clarify the immediate effects of leg press-based coordination training on ankle sway in individuals with CAI. In individuals with CAI, coordination training with a leg press immediately produced a significant group × time interaction in average ankle angular jerk cost during the anterior and posterior medial reach, and the CT group showed a decrease in average ankle angular jerk cost post-intervention. In addition, although no interaction occurred, symmetrical pre- and post-intervention differences in the ankle average angular jerk cost during the posterolateral reach also occurred between the two groups. Therefore, the results support this hypothesis and suggest that coordination training with a leg press device reduces ankle sway compared with regular leg-press training. Previous studies have reported improvements in muscle coordination and postural control after coordination training in individuals [8,21,22]. Furthermore, a single session of balance training for patients with CAI has been reported to increase spinal reflex modulation and corticospinal excitability of the soleus muscle [7]. In the present study, ankle sway in the YBT was reduced by changes in the muscle group recruitment pattern, reaction time, and muscle coordination ability associated with the above. However, no improvement in ankle inversion proprioception (absolute error and variable error in PJRS) was observed in the coordination training group. Therefore, the reduction in ankle sway in the present study may not have been caused by an improvement in proprioception in the direction of inversion. Sensory information is important in motor control because it is used to adjust muscle strength [23]. One explanation for this discrepancy is that although the tests used in this study to assess proprioception have been used in previous studies [19], the primary assessment was joint position sense. In proprioception, muscle spindles for joint position sense and Golgi tendon organs for force sense are the main receptors, and they may be independent of each other [24]. In particular, the passive joint repositioning test used in this study does not reflect sensations caused by muscle contraction, such as force sense, because no muscle activity is absent. However, force sense may be particularly important in muscle dysregulation in individuals with CAI [21]. Therefore, the mechanism of this result needs to be verified by examining other elements of proprioception and recruitment patterns of muscle groups in the future.

However, the ankle sway increased after training in the group with regular leg-press training, which was set up as the CON group. Leg presses are generally used to train hip and knee extensors. Individuals with CAI control their posture by compensating for ankle instability with the hip joint [25], and hip training in a situation where regular leg-press training did not require ankle control may result in dominant hip control after training. This may be related to the fact that the CON group showed an opposite trend in hip flexion angle during anterior reach, with the CON group showing a larger hip flexion angle and the CT group a smaller hip flexion angle. However, this point alone may be difficult to explain, considering other reach directions and motions. Because only the motion angle was measured in this study, the muscle activity and ground reaction force were not verified, and the clarification of the mechanism is a future issue.

In ankle sprains, slight motion in the direction of ankle inversion or adduction is the starting point of injury [26], and excessive joint motion leads to the development of ankle instability by causing tissue microdamage [3,27]. Increased ankle instability leads to decreased activity [28] and increased load on other parts of the body [25,29], as well as an increased risk of developing pain and ankle osteoarthritis. Therefore, interventions to reduce ankle joint sway are important. These findings highlight the importance of coordination training prior to competitions or high-level training. Coordination training is reportedly effective in preventing recurrent sprains in individuals with a history of ankle sprains [30]. However, verifying whether the training conducted in this study led to injury prevention was not possible, and this issue needs to be investigated in the future.

Limitations

A limitation of the study is that it examined the immediate effects of coordination training using leg presses as a warm-up, such as before playing. However, the long-term effects of coordination training have not been tested. Although previous studies [8-10] have shown the long-term effects of ankle coordination training on postural balance, none have examined ankle sway. Therefore, the long-term effects of leg-press-based coordination training on ankle sway need to be verified. Furthermore, regarding the sample size, 14 persons in each group were needed for calculation purposes; however, the number of persons meeting the inclusion criteria for this study in the team that was requested to collaborate was insufficient. β-errors may have occurred, which may have affected the secondary outcomes in particular.

## Conclusions

This study investigated whether coordination with a leg press has an immediate effect on ankle sway in patients with CAI. Leg-press coordination training was effective in reducing ankle sway during dynamic tasks in individuals with CAI.
